# Prevalence and variability of siderophore production in the *Achromobacter* genus

**DOI:** 10.1128/spectrum.02953-23

**Published:** 2024-02-05

**Authors:** P. Sorlin, E. Brivet, V. Jean-Pierre, F. Aujoulat, A. Besse, C. Dupont, R. Chiron, E. Jumas-Bilak, Q. Menetrey, H. Marchandin

**Affiliations:** 1 HydroSciences Montpellier, Université de Montpellier, CNRS, IRD, Montpellier, France; 2 Service de Microbiologie et d’Hygiène hospitalière, CHU de Nîmes, Nîmes, France; 3 Laboratoire de Bactériologie, CHU de Montpellier, Montpellier, France; 4 Centre de Ressources et de Compétences de la Mucoviscidose, CHU de Montpellier, Montpellier, France; 5 Laboratoire d’Écologie Microbienne Hospitalière, CHU de Montpellier, Montpellier, France; 6 INFINITE—Institute for Translational Research in Inflammation, Université de Lille, INSERM U1286, CHU Lille, Lille, France; Universidad Nacional Autonoma de Mexico - Campus Morelos, Cuernavaca, Mexico

**Keywords:** cystic fibrosis, opportunistic pathogen, *Achromobacter xylosoxidans*, *Achromobacter* spp., siderophores

## Abstract

*Achromobacter* spp. are opportunistic pathogens of environmental origin increasingly isolated in patients with underlying conditions like cystic fibrosis (CF). Despite recent advances, their virulence factors remain incompletely studied, and siderophore production has not yet been investigated in this genus. The aim of this study was to evaluate the production of siderophores in a large collection of *Achromobacter* spp. and evaluate the variability according to the origin of the strain and species. A total of 163 strains were studied, including 128 clinical strains (CF and non-CF patients) and 35 strains of environmental origin. Siderophores were quantified by the liquid chrome azurol-sulphonate assay. Species were identified by *nrdA* gene-based phylogeny. Strains were assigned to 20 species, with *Achromobacter xylosoxidans* being the most represented (51.5% of strains). Siderophore production was observed in 72.4% of the strains, with amounts ranging from 10.1% to 90% siderophore units. A significantly higher prevalence of siderophore-producing strains and greater production of siderophores were observed for clinical strains compared with strains of environmental origin. Highly variable observations were made according to species: *A. xylosoxidans* presented unique characteristics (one of the highest prevalence of producing strains and highest amounts produced, particularly by CF strains). Siderophores are important factors for bacterial growth commonly produced by members of the *Achromobacter* genus. The significance of the observations made during this study must be further investigated. Indeed, the differences observed according to species and the origin of strains suggest that siderophores may represent important determinants of the pathophysiology of *Achromobacter* spp. infections and also contribute to the particular epidemiological success of *A. xylosoxidans* in human infections.

**IMPORTANCE:**

*Achromobacter* spp. are recognized as emerging opportunistic pathogens in humans with various underlying diseases, including cystic fibrosis (CF). Although their pathophysiological traits are increasingly studied, their virulence factors remain incompletely described. Particularly, siderophores that represent important factors of bacterial growth have not yet been studied in this genus. A population-based study was performed to explore the ability of members of the *Achromobacter* genus to produce siderophores, both overall and in relevant subgroups (*Achromobacter* species; strain origin, either clinical—from CF or non-CF patients—or environmental). This study provides original data showing that siderophore production is a common trait of *Achromobacter* strains, particularly observed among clinical strains. The major species, *Achromobacter xylosoxidans*, encompassed both one of the highest prevalence of siderophore-producing strains and strains producing the largest amounts of siderophores, particularly observed for CF strains. These observations may represent additional advantages accounting for the epidemiological success of this species.

## INTRODUCTION

Bacteria of the *Achromobacter* genus are Gram-negative bacilli present in diverse environments, including hospital, domestic, and outdoor environments, mostly soil and water (1–3). In soil, *Achromobacter* spp. are recognized as plant-growth-promoting rhizobacteria playing critical roles in plant growth and development (4); they are also considered as biocontrol agents and remediating microorganisms of polluted soils contaminated by oils, heavy metals, polycyclic aromatic hydrocarbons, etc. (5). *Achromobacter* spp. also act as opportunistic pathogens in humans, including patients with cystic fibrosis (CF). In this autosomal recessive genetic disease, dehydration and thickening of mucus in the airways result in impaired mucociliary clearance, microbial stasis, and repeated microbial infections (6, 7). Besides *Pseudomonas aeruginosa*, the most frequent pathogen of environmental (ENV) origin in this context, *Achromobacter* spp. have been recognized as emerging pathogens with a colonization rate that has increased throughout the world, like in France where 6.4% of CF patients were colonized in 2021 versus 3.7% in 2006, until the implementation of CF transmembrane conductance regulator corrector and potentiator combinations therapies (8). *Achromobacter* spp. are also agents of healthcare-associated infections and are involved in various infectious processes in immunocompromised patients, for example, in onco-hematology patients presenting bacteremia and catheter-related infections (9, 10). More rarely, infections have also been described in immunocompetent patients (11). These observations have long given the *Achromobacter* spp. their reputation as pathogens of mild virulence. However, more recently, clinical studies have provided evidence on the pathogeny and clinical impact on the pulmonary function of *Achromobacter* spp. in CF patients (12–14). On the other hand, laboratory studies have shown that *Achromobacter* strains share important pathophysiological characteristics and virulence factors with other major pathogens of environmental origin like *P. aeruginosa*, such as multidrug resistance, biofilm formation, and high inflammatory properties (15–17). Despite these advances, some major bacterial virulence factors remain largely unexplored in the *Achromobacter* genus as is the case for siderophores. Siderophores are high-affinity, low-molecular-weight molecules that bind to extracellular iron and facilitate its acquisition by bacteria (18–20). As iron is an essential nutrient for bacterial survival and growth, the ability to produce siderophores provides an advantage for pathogenic bacteria by allowing them to acquire the available iron in the host’s environment. This makes siderophores key determinants of bacterial virulence. Currently, over 500 bacterial siderophores have been characterized (21, 22) and classified according to their chemical structure (iron-binding moieties) into the families of catecholates, hydroxamates, phenolates, and carboxylates and an additional category known as “mixed-type” siderophores (23–26). Among them, catecholates, which include, for example, the enterobactin found in *Escherichia coli* strains (25), have been described as having the highest iron affinity (23, 25). Siderophores are produced by all the major human pathogens, but so far, the production of siderophores by members of the *Achromobacter* genus has received little attention and has only been reported in a few studies, each referring to a single strain, mostly of environmental origin (27–32). On another hand, genes encoding siderophores or siderophore receptors have already been identified in whole genome sequences of clinical strains (33, 34). However, beyond gene content, evaluating the real factors produced by these bacteria is an important contribution to elucidating the pathogeny of the bacterium.

Finally, the members of the *Achromobacter* genus are divided into 21 species but are more usually considered as a whole, with no specific data available for individual species accurately identified. Indeed, species identification in this genus is challenging in routine practice despite the optimized mass spectrometry database recently proposed although not yet widely available (35). Most routine tools are still unable to accurately distinguish species. Consequently, molecular tools are required for unquestionable species identification, and these are essential for recognizing the specific characteristics of individual species. In studies based on accurate species identification, *A. xylosoxidans* appears to be the most commonly identified species in clinical settings, suggesting that some species may have selective advantages in their ability to infect humans (1, 36).

In this context, the aim of this study was to describe siderophore production among a large collection of clinically and genetically documented *Achromobacter* strains to evaluate the potential importance of these factors in the pathophysiology of *Achromobacter* spp. infections. To do so, siderophore production was studied both qualitatively (prevalence of producing strains) and quantitatively (amounts of siderophores produced). The results obtained for the overall strain collection provide first insights into the global ability of *Achromobacter* spp. to produce iron-chelating molecules. An analysis of subgroups of strains made it possible to identify specific characteristics in siderophore production according to (i) the species, as identified by *nrdA* gene sequencing and *nrdA* gene-based phylogeny, and (ii) the origin of the strains, by comparing results obtained for strains of environmental origin to those of strains of clinical origin, either from CF or non-CF (NCF) patients.

This study was the first to evaluate siderophore production in a large collection of *Achromobacter* strains encompassing different species and isolation sources. It, therefore, provides original data on the topic, showing that siderophore production is a common trait of *Achromobacter* strains but that variability is observed among species and according to the origin of the strain. Siderophores must be added to the panel of virulence factors that might be produced by *Achromobacter* spp. and are probably important determinants of the epidemiological success of members of this genus in human infections.

## RESULTS

### Genetic and species diversity within the collection of *Achromobacter* strains studied

**Fig 1:**
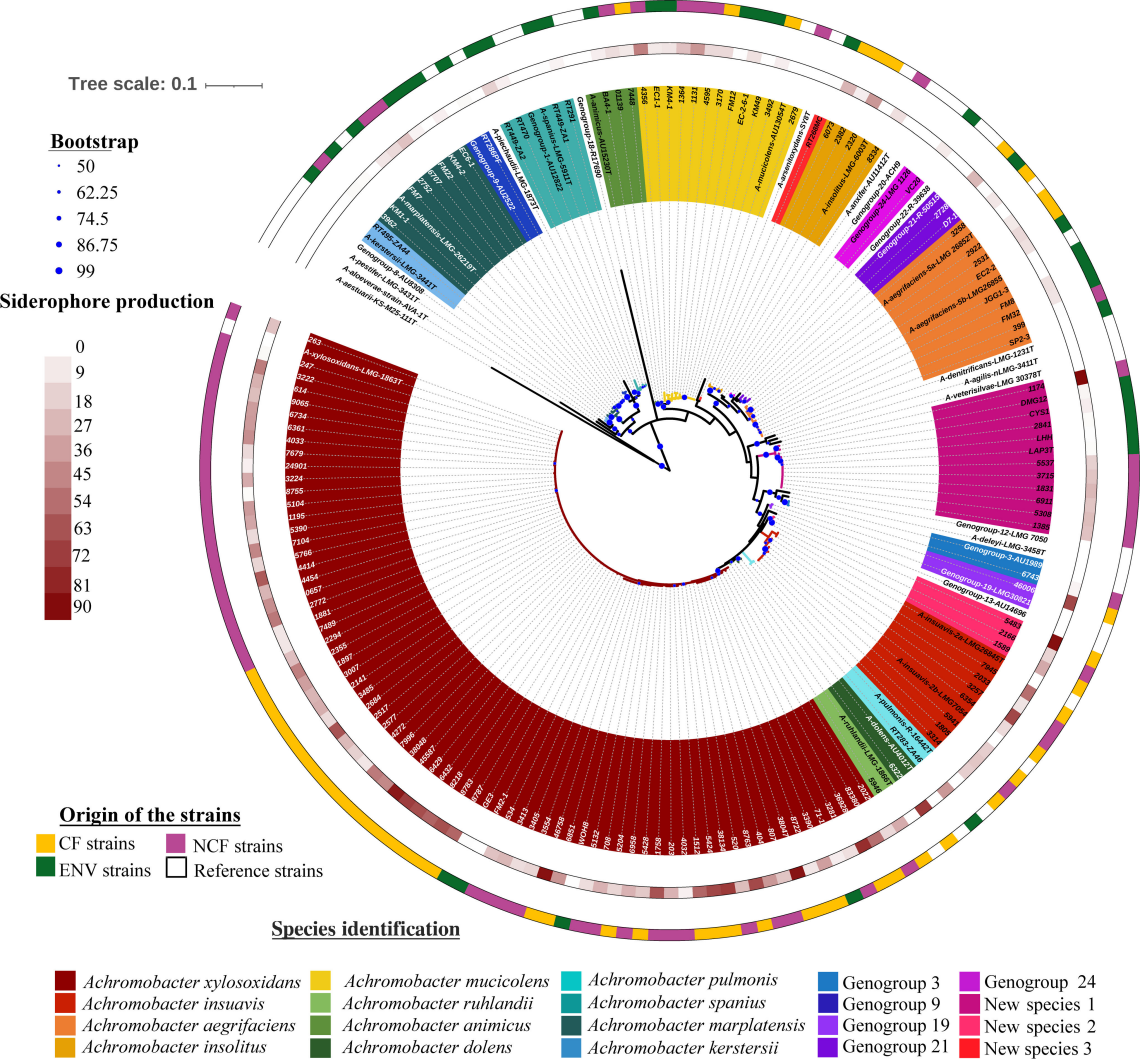
Maximum-likelihood tree based on *nrdA* partial sequence (765 bp) indicating the relative placement of the 163 *Achromobacter* spp. strains according to their siderophore production and origin (CF, NCF, and ENV). Branches are colored according to species affiliation. The outer ring depicts the strains’ origins: CF clinical strains (yellow), NCF clinical strains (pink), and strains of environmental origin (green). The inner ring represents siderophore production by a color gradient from white (no or low production) to red (high production) according to percent siderophore unit (psu) measured by the liquid chrome azurol-sulphonate assay (0–90 psu). The scale bar indicates the number of substitutions per nucleotide position. Blue circles at the nodes are support values estimated with 100 bootstrap replicates. The figure was constructed using iTOL.

A total of 70 alleles of the *nrdA* gene were found for the 163 strains studied (Table S1) and assigned to 20 species by *nrdA*-gene-based phylogeny (Fig. 1), either to species with validly published names (*n* = 12) or not (*n* = 5 genogroups) or hitherto uncharacterized species (*n* = 3) (Table 1). *A. xylosoxidans* was the most widely represented species in the collection (51.5%, 84 strains). Twelve species included less than four strains, of which eight species comprised a single strain. Heterogeneity in the distribution of the 163 strains and the 20 species according to the source of their recovery is presented in Table 1. *A. xylosoxidans* was one of the four species identified in the three studied groups [CF, NCF, and ENV], together with *Achromobacter aegrifaciens, Achromobacter animicus,* and *Achromobacter mucicolens*; other species being identified in two (*n* = 6) or only one (*n* = 10) of these three study groups (Table 1). However, *A. xylosoxidans* was the main species identified among clinical strains, either from CF or NCF patients. The three subgroups of strains, CF, NCF, and ENV, also displayed distinct species diversity, but the greatest species diversity was found in the ENV subgroup (13 species, 29 *nrdA* alleles) despite being the smallest (35 strains) compared with the two groups of clinical isolates (CF: 67 strains belonging to 11 species, 32 *nrdA* alleles; NCF: 61 strains belonging to 10 species, 27 *nrdA* alleles).

**TABLE 1:** Distribution of the 163 *Achromobacter* spp. strains of this study according to species identified by *nrdA* gene-based phylogeny and origin

*Achromobacter* species (*n* = 20)^*a*^	Clinical strains (*n* = 128)		Strains of environmental origin	Total
	CF	NCF		
*A. xylosoxidans*	42	38	4	84
*A. insuavis*	9	3	–	12
*A. mucicolens*	2	5	5	12
New species 1	–	7	5	12
*A. aegrifaciens*	3	1	5	9
*A. marplatensis*	–	3	5	8
*A. insolitus*	3	1	–	4
*A. spanius*	–	–	4	4
*A. animicus*	1	1	1	3
New species 2	2	1	–	3
*A. ruhlandii*	2	–	–	2
Genogroup 21	1	–	1	2
*A. dolens*	1	–	–	1
*A. kerstersii*	–	–	1	1
*A. pulmonis*	–	–	1	1
Genogroup 3	–	1	–	1
Genogroup 9	–	–	1	1
Genogroup 19	1	–	–	1
Genogroup 24	–	–	1	1
New species 3	–	–	1	1
Total	67	61	35	163

^*a*^ Species are presented in descending order according to the total number of strains. CF, strain(s) from patient(s) with cystic fibrosis. NCF, strain(s) from other patient(s) not suffering from cystic fibrosis, –, no isolate of this species or from this origin.

### Siderophore production is common in the *Achromobacter* genus, mostly observed for clinical strains

**Fig 2:**
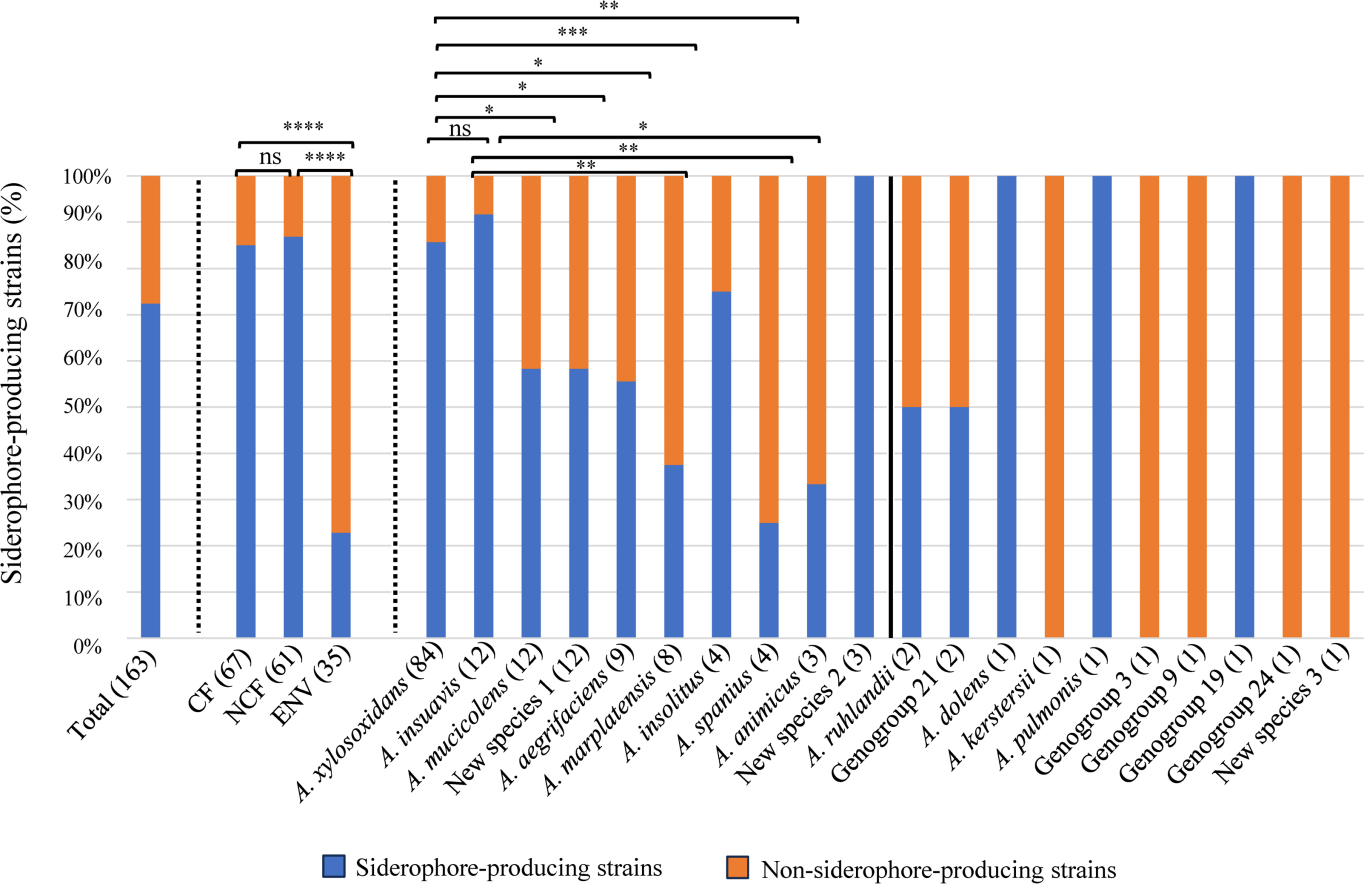
Relative proportion (%) of siderophore-producing *Achromobacter* strains in the overall population (left), according to origin (CF, NCF, and ENV) and species (right). Species are ranked by the number of strains, indicated in brackets. A vertical line separates species represented by one or two strains only. Siderophore-producing strains are shown in blue and non-producing strains in orange. The Chi-squared test was used to compare the prevalence of siderophore-producing strains according to origin (CF, NCF, and ENV) or species. Mann-Whitney test was used to compare the prevalence of siderophore-producing strains according to the species. *P < 0.05; **P < 0.01; ***P < 0.001; ****P < 0.0001; and ns: not significant (indicated when strain numbers in compared groups were enough to test significance only).

Among the 163 strains studied, 118 (72.4%) were able to produce siderophores under the experimental conditions of the study. Significantly higher proportions of siderophore-producing strains were observed among clinical strains, either from CF patients (85.1%, 57/67) or NCF patients (86.9%, 53/61) compared with strains of environmental origin (22.8%, 8/35) (*P*-values *<* 0.0001) (Fig. 2). Looking specifically at the 118 siderophore-producing strains, these were mostly clinical strains with 57 (48.3%) of these strains originating from CF patients and 53 from non-CF patients (44.9%), whereas only 8 were of environmental origin (6.7%) (Table S2). Based on these results, all 35 strains of environmental origin were tested again for siderophore production in the same conditions except for the growth temperature that was lowered to 30°C. Similar results were obtained with a mean difference of 0.7 percent siderophore unit (psu) (0–2.9) between both growth conditions (37°C or 30°C), which did not affect the strain categorization as siderophore producer or non-siderophore producer (*P*-value > 0.5) (Table S3). Among the eight strains of environmental origin that produced siderophores, six were recovered from the domestic environment of patients with CF (13 other strains from the domestic environment of patients with CF were considered non-producers), and the two remaining strains were isolated from wastewater and soil samples, respectively (Table S3).

### Siderophore production is differentially observed according to species

Overall, the ability to produce siderophores was observed for strains belonging to 15 out of the 20 species identified in this study since siderophore production was not detected for strains assigned to *Achromobacter kerstersii*, genogroup 3, genogroup 9, genogroup 24, or to new species 3. However, this observation is likely to be biased by the single strain analyzed for each of these five species. By contrast, siderophore production was observed for species represented by single strains like those belonging to *Achromobacter dolens, Achromobacter pulmonis,* and genogroup 19, or very few strains (<4) like the three clinical strains of new species 2, which were all siderophore producers (Fig. 2). Regarding the eight species represented by four strains or more, the prevalence of siderophore producers varied from 25% for *Achromobacter spanius* to 91.7% for *Achromobacter insuavis* strains. In addition to being the most highly represented species, *A. xylosoxidans* also displayed a high proportion of 85.7% (72/84) siderophore-producing strains, significantly higher than for *A. mucicolens*, new species 1, *A. aegrifaciens* (*P*-value < 0.05), *A. spanius* (*P*-value < 0.01), and *A. marplatensis* (*P*-value < 0.001) (Fig. 2). The species *A. insuavis* also included a high proportion of siderophore-producing strains, which was significantly higher than for *A. marplatensis* and *A. spanius* (*P*-values < 0.01), and *A. animicus* (*P*-value *<* 0.05).

**Fig 3:**
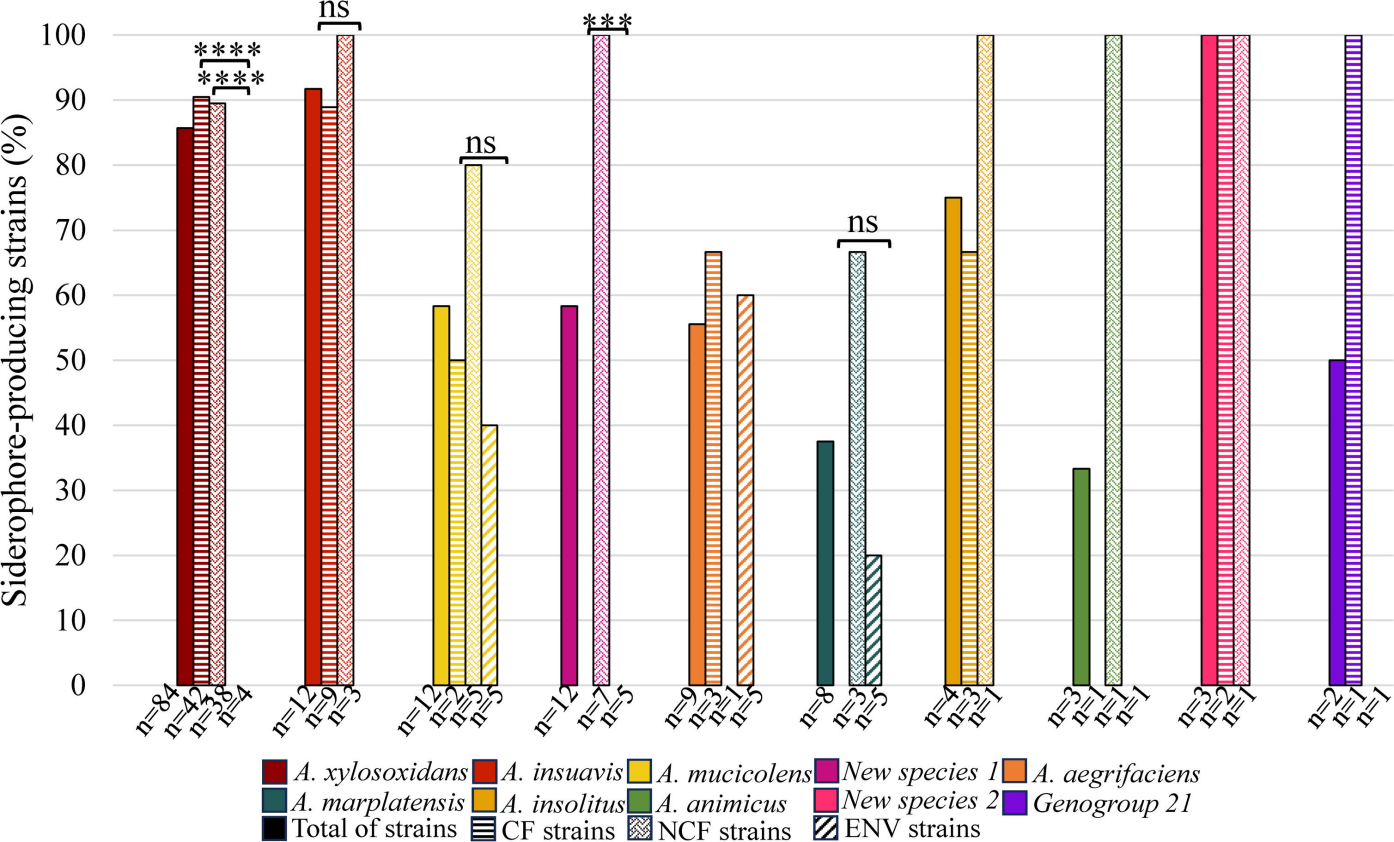
Proportion of siderophore-producing strains depending on *Achromobacter* species and strain origin. Each species containing siderophore-producing strains is represented by a color code. Species that do not contain any siderophore-producing strain (5/20) or that contain strain(s) of a single origin (5/20) are not included. CF strains are indicated by a horizontal striped pattern, NCF strains by a solid dotted color pattern, and ENV strains by a striped pattern with diagonally oriented bands; total for each species is represented by a solid color. For species in which siderophore-producing strains originated from only two of the three origins (CF, NCF, and ENV) while no strains originating from the third origin produced siderophores, comparative results are shown for strains of the three origins, resulting in the absence of some histograms although a number of strains are indicated (e.g., none of the four *A*. *xylosoxidans* strains of environmental origin produced siderophores). The Chi-squared test was used to test for significance. *P < 0.05; **P < 0.01; and ns: not significant (indicated when strain numbers in compared groups were enough to test significance only).

When looking at the proportion of siderophore-producing strains according to strain origin within each species, several differences were observed according to the clinical or environmental origin of the strains or the CF or NCF origin within clinical strains (Fig. 3). For most cases, strain numbers were too low to test statistical significance and draw robust conclusions, but for *A. xylosoxidans* and new species 1, percentages of siderophore-producing strains were significantly higher among clinical strains than among strains of environmental origin (*P*-values < 0.0001 and < 0.01, respectively) (Fig. 3).

### Amounts of siderophore produced are variable according to strain origin and species

The 118 strains able to produce siderophores generated an average amount (±standard deviation) of 33.4 ± 21 psu (range: 10.1–91) (Table S2). Variability in results obtained for each strain (inferred from the psu of two biological replicates each consisting of two technical replicates) did not exceed 5.3 psu, indicating good reproducibility, as observed for strain *Pa* IV33 used as the positive control that produced 84.04 ± 2.5 psu (calculated from 54 measurements).

**Fig 4:**
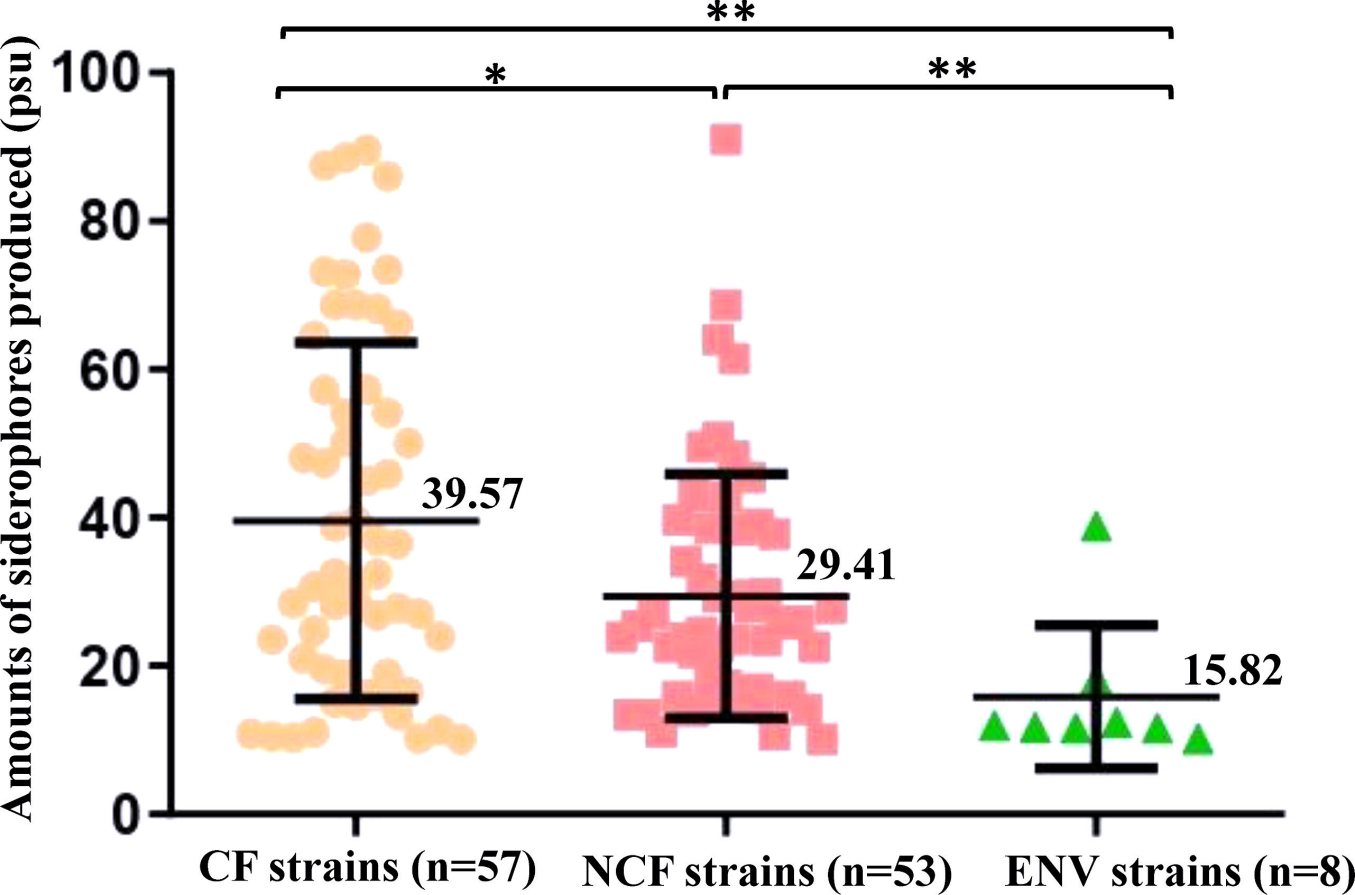
Amounts of siderophores produced by *Achromobacter* spp. according to the origin of strains. Results are expressed as percent siderophore units (psu). Each point represents the mean value of four measures for a siderophore-producing strain (*n* = 118). Orange: strains isolated from patients with CF; pink, strains isolated from patients not suffering from CF; and green, strains of environmental origin. Mean psu values and standard deviations are indicated for each group. The Mann-Whitney test was used to test for significance. *P < 0.05 and **P < 0.01.

Depending on the origin of the strain, the average siderophore production was 39.6 ± 24 psu for CF strains (range: 10.1–89.6), 29.4 ± 16.5 psu for NCF strains (range: 10.1–91), and 15.8 ± 9.6 psu for ENV strains (range: 10.2–38.9) (Fig. 4). Differences observed between these subgroups of strains were all statistically significant (Fig. 4). Clinical strains, either from CF or NCF patients, produced significantly more siderophores than environmental strains (*P*-values < 0.01), and the ENV group of strains was also the least heterogeneous in terms of levels of siderophore produced. Among all clinical strains, CF strains produced significantly higher amounts of siderophores than NCF strains (*P*-value < 0.05) (Fig. 4).

**Fig 5:**
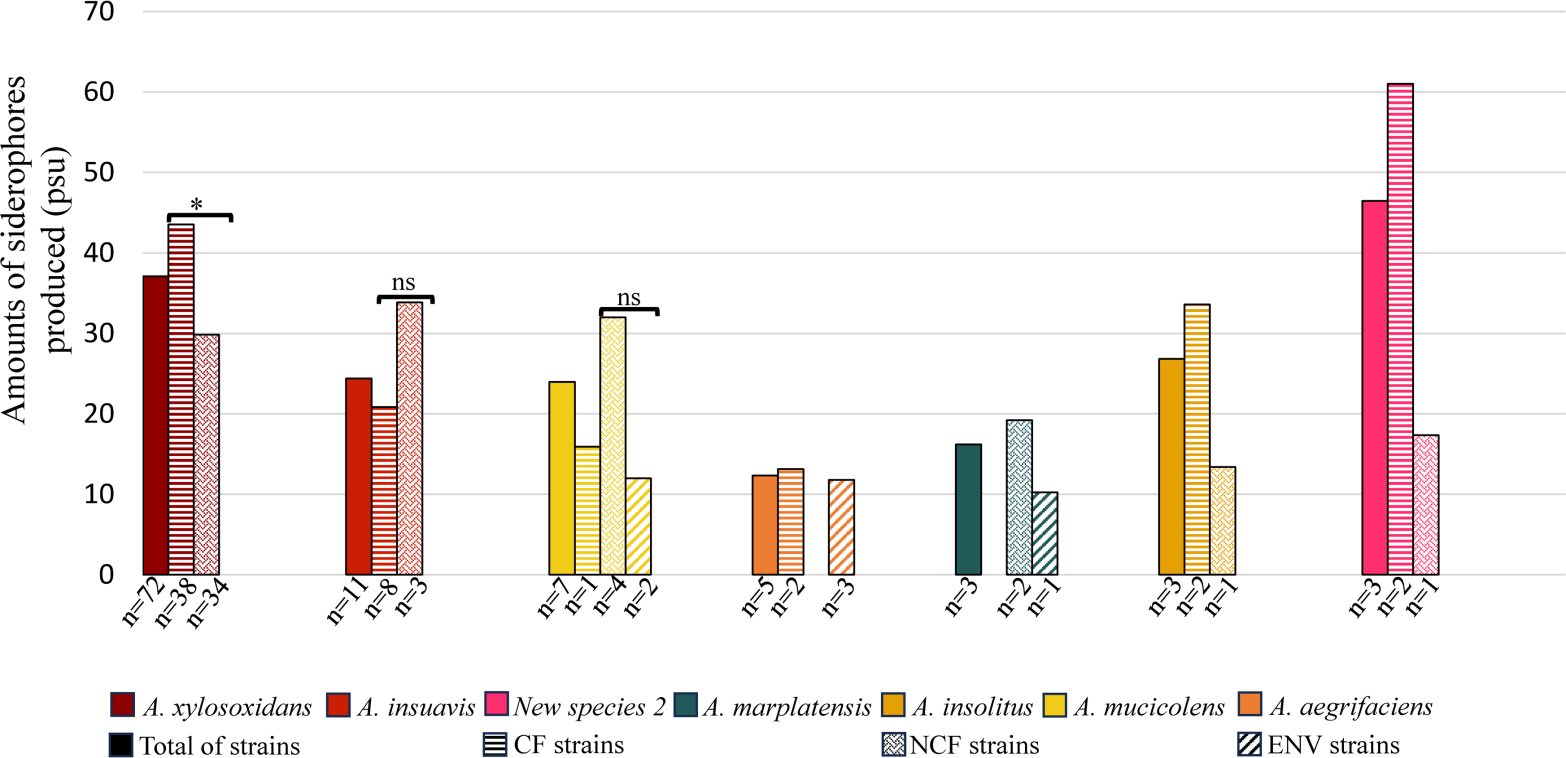
Amounts of siderophores produced by *Achromobacter* spp. according to species and origin. Each species containing siderophore-producing strains is represented by a color code. Species that do not contain any siderophore-producing strain (5/20) or that contain siderophore-producing strain(s) from a single origin (8/20) are not included. CF strains are indicated by a horizontal striped pattern, NCF strains by a solid dotted color pattern, and ENV strains by a striped pattern with diagonally oriented bands; total for each species is represented by a solid color. The Mann-Whitney test was used to test for significance. **P < 0.01 and ns: not significant (indicated when strain numbers in compared groups were enough to test for significance only).

An interesting observation was made for five clinical strains that produced high levels of siderophores of about 90 psu, surpassing that of strain *Pa* IV33. These five strains included four strains isolated from CF patients (three *A. xylosoxidans* of distinct *nrdA* genotype and one strain assigned to new species 2) and one strain belonging to new species 1 and recovered from an implantable device in an NCF patient (Table S3). At the species level, the sole representative of *A. dolens* and genogroup 19 produced high amounts of siderophores: 73.2 and 68.7 psu, respectively (Table S2). Species represented by more than one strain produced mean amounts of siderophores ranging from 12.3 psu for the nine *A. aegrifaciens* strains to 46.5 psu for the three strains belonging to new species 2 (Fig. 5). The mean amount of siderophores produced by the major species *A. xylosoxidans* was 37.1 psu (range: 10.1–88.5). This was significantly more than the amounts of siderophores produced by *A. insuavis* (*P*-value < 0.05), *A. aegrifaciens* (*P*-value < 0.001), and *A. marplatensis* (*P*-value < 0.05). Similarly, members of both new species 1 and new species 2 produced significantly more siderophores than *A. aegrifaciens* (*P*-values < 0.05) (Table S2).

A focus was then made on the amounts of siderophores produced according to the origin of strains within each species. Several differences were observed with greater amounts of siderophores being produced by clinical strains than by strains of environmental origin for the three species (*A. mucicolens*, *A. aegrifaciens,* and *A. marplatensis*) including strains of both origins. However, none of these differences were considered significant, either due to too low numbers of strains to test statistical significance (*A. aegrifaciens* and *A. marplatensis*) or to a non-significant *P*-value > 0.05 (*A. mucicolens*). Among the clinical strains, distinct levels of siderophores were produced, being either higher for CF strains compared with NCF strains (*A. xylosoxidans*, *A. aegrifaciens*, *A. insolitus*, and new species 2) or, on the contrary, lower (*A. insuavis* and *A. mucicolens*), but differences were only statistically significant for *A. xylosoxidans* (*P*-value < 0.05) (Fig. 5).

### Siderophore production is not correlated to growth differences in iron-depleted minimal medium 9

Because results of siderophore dosage were not normalized according to growth in iron-depleted minimal medium 9 (MM9) in this study and therefore might have been influenced by the strain’s ability to grow in this minimal medium, we analyzed growth curves for a selection of 33 strains representative of strain origin (8 from CF patients, 11 from NCF patients, and 14 from environmental origins) and species (Table S3). Variable growth in MM9 was observed depending on the strain considered (Table S3; Fig. S1). However, the Spearman’s correlation run to determine the relationship between the 33 psu and optical density (OD)_600nm_ values showed that there was no correlation between the amount of siderophores produced and the growth in MM9 (*r* = 0.159 and *P* = 0.375), thereby excluding that differences of growth in MM9 accounted for the differences observed in siderophore production in this study (Fig. S1).

## DISCUSSION

### Siderophores are yet little studied among the panel of virulence determinants of *Achromobacter* spp.

Based on their involvement in various infections in both CF and NCF patients, *Achromobacter* spp. have received increasing interest with regard to specifying their pathogenicity and deciphering the associated molecular determinants. Laboratory findings have progressively described a series of abilities and virulence determinants related to clinical observations (16). *Achromobacter* spp. displayed numerous pathogenic phenotypes contributing to host colonization and persistence through adhesion, motility, biofilm formation, drug and multidrug resistance, high survival ability in biocides, and the secretion of diverse tissue-degrading enzymes and toxins (15, 37–43). They also possess secretion systems well-known for contributing to virulence in many pathogens (44–46). Pro-inflammatory power was demonstrated both *in vitro* and *in vivo* (47–50) showing that *Achromobacter* spp. are capable of inducing inflammation in a similar way to *P. aeruginosa* (48, 50). Genetic and genomic studies support the aforementioned observations showing that these bacteria have a relatively large genome size (6–7 Mb) and are capable of hypermutation and that genetically diversified populations generated through adaptation processes are present during persistent colonization. All these characteristics represent important traits involved in host adaptation and virulence, all contributing to the success of *Achromobacter* spp. in human infections (9, 16, 37, 51, 52). In addition, comparative genomics reveals potential genetic determinants facilitating adaptation to a pathogenic lifestyle in humans, as they have been observed in strains from CF patients while absent in strains of environmental origin (33). Considering all the acquired knowledge on the pathogenicity of *Achromobacter* spp., they are now considered surreptitious opportunistic pathogens (53).

Contrasting with this increasing characterization of virulence determinants, siderophores have not been the subject of any specific studies to date in the *Achromobacter* genus. Strikingly, a recent large-scale genome study on virulence gene content of 101 *Achromobacter* spp. strains did not mention any factors related to iron uptake/siderophore secretion pathways (36). Scarce data currently available are limited to six studies, each referring to a single strain of *Achromobacter* sp., mostly of environmental origin (27–32). However, each provides data on the ability to produce siderophores or on gene content consistent with siderophore biosynthesis for the studied strains. Tamariz-Angeles et al. reported a plant-associated strain (BEP19-Dm) from heavy metal-polluted rhizospheres in Peru, displaying the ability to produce siderophores, as observed by Jana et al. for strain SQU-1 isolated from date palm rhizosphere. Identified as *A. marplatensis* and *A. xylosoxidans,* respectively, these identifications must, however, be regarded with caution as a non-discriminatory method, i.e., 16S rRNA gene sequencing, was used (31, 32). Another strain, *Achromobacter* sp. RZS2, isolated from groundnut rhizosphere in India was shown to be able to produce a large amount of siderophores (92.61 psu) (28), as observed for some of the clinical strains in the present study. Other studies showed that various types of siderophores might be produced in the *Achromobacter* genus. *Achromobacter* sp. strain MM1 of environmental origin, isolated from *Fusarium* suppressive soil in Italy, was shown to produce a hydroxamate-type siderophore (27), while strain KAs 3-5T, representative of a new *Achromobacter* species and isolated from groundwater in Bengal, secreted a carboxylate-type siderophore called achromobactin, already identified in *Pseudomonas syringae* (30, 54). In the latter study, the authors gave complementary information on the siderophore pathway in strain KAs 3-5T from whole genome sequence analysis. They showed that the strain might be able to acquire and store iron through a ferrous transport system similar to that of *P. aeruginosa* (55), including a TonB-dependent siderophore receptor, an ABC-type iron transporter, a ferric uptake regulation protein, a ferrichrome iron receptor, a periplasmic Fe-binding protein, and putative Fe reductases (30). Another unique study included a clinical strain (AXX-A) recovered from a CF patient and identified as belonging to *A. insuavis* although initially reported as an *A. xylosoxidans* strain (33). Data inferred from whole genome sequence analysis of strain AXX-A showed unique genes related to iron transport and utilization and a set of nine genes involved in the biosynthesis of a hydroxamate siderophore identical to alcaligin, a high-affinity iron chelating agent produced by *Alcaligenes denitrificans* (now *Achromobacter denitrificans*), *Bordetella pertussis,* and *Bordetella bronchiseptica*, suggesting that *Achromobacter* spp. may be able to overcome iron-limited conditions (33, 56, 57).

Based on the literature reviewed, it appears that siderophore production by members of the *Achromobacter* genus has been little investigated and warrants further studies.

### Unique insights into siderophore production by *Achromobacter* spp. by studying a large collection of accurately identified strains from diverse origins

In this context, siderophore production was studied in a large collection of *Achromobacter* strains originating from various sources, either clinical or environmental. As routine phenotypic tools and 16S rRNA gene sequencing are inappropriate for accurate species assignation, strains were identified based on a molecular-based method previously recognized as being powerful and discriminatory in the *Achromobacter* genus, the *nrdA* gene sequence analysis (58). This approach also allowed us to describe the genetic diversity of both the overall population and the different subgroups considered in this study (species and origin). In congruence with previous studies on clinical strains, *A. xylosoxidans* was the most frequently identified species, either in CF or NCF patients (41, 59). However, nearly half the patients were colonized by strains of other species, currently far less studied than *A. xylosoxidans* and most of them shared the ability to produce siderophores with *A. xylosoxidans*. By contrast, strains of environmental origin were more homogeneously distributed in several species in this study. Few studies assessed the relative importance of *Achromobacter* species in the environment, but a recent study including 53 environmental strains from domestic, hospital, and natural environments also identified a variety of species although not strictly identical to that reported herein (35). Representatives of three new *Achromobacter* species were present in the studied collection, suggesting a high, still under-described, genetic diversity in the *Achromobacter* genus, as previously observed (36). A variable species and genetic diversity was observed in the subgroups of strains according to origin in our study, the strains of environmental origin being more diverse than clinical strains, either from CF or NCF patients, suggesting that not all strains of environmental origin might be able to infect humans and, in some cases, persist through adaptation to specific anatomical niches. In these cases, the selective advantages that might be presented by certain strains are crucial for strain survival under local selective biotic and abiotic pressures. Among the numerous pathophysiological traits of *Achromobacter* spp. presented above, the ability to produce siderophores thus appeared an important determinant to be studied. Indeed, siderophores not only represent well-established factors involved in bacterial virulence but they also might be involved in competitive interactions between *Achromobacter* sp. and other microorganisms and in modulation of host cellular pathways (19, 60, 61).

The population-based study conducted here revealed unprecedented findings on siderophore production in the *Achromobacter* genus. First of all, a large majority of strains were found to be able to produce siderophores under the conditions of the study. However, for certain species, too small numbers of strains prevented any robust interpretation, and the results must still be complemented by collecting and studying additional strains, particularly for the five species with single strains included and no siderophore-producing representatives. On the other hand, several observations distinguished strains according to their origin, regarding their capacity to produce siderophores. Overall, clinical strains were significantly more likely to produce siderophores than strains of environmental origin, and they also produced significantly higher amounts of siderophores. These findings were independent of both the temperature used for the growth of strains from environmental origin and the growth in MM9. Therefore, the results of this study suggested that clinical strains might have distinct iron requirements than environmental strains. As these results may reflect specific traits of strains recovered from human clinical samples or be affected by the distinct relative importance of species between study groups, the results obtained for each species represented by more than one strain in at least two of the three groups of the study (CF, NCF, and ENV) were further examined. *A. xylosoxidans*, which is the major species in this study and is also reported as the major species identified from human clinical samples, combined one of the highest proportions of siderophore-producing strains and one of the highest amounts of siderophores produced, particularly observed for clinical strains, and among clinical strains, for CF strains. Literature remains poor regarding this kind of comparative analysis on siderophore production according to species within a genus or origin of strain. Including a small number of *P. aeruginosa* strains, Ali and Vidhale showed that clinical strains produced higher amounts of siderophores than strains of environmental origin (62). In other distantly related genera, similar observations were made for *Aeromonas hydrophila* by Naidu et al., comparing 19 strains from human diarrhea samples and 11 strains from freshwater ponds (63). In their study, siderophore production under iron-limiting conditions was significantly influenced by the origin of the strains. Indeed, they found that clinical strains consistently produced higher levels of siderophores than environmental strains (63). Although most microorganisms are thought to secrete siderophores when facing iron stress, another interesting finding in this study was that, unlike the environmental strains, most of the clinical strains also produced considerable amounts of siderophores under iron-rich conditions, again suggesting that clinical strains and their environmental counterparts may have distinct iron requirements. Beyond the global amount of siderophores produced, different siderophores with various degrees of iron affinity can also be produced by a species; for example, *P. aeruginosa* secretes pyoverdine (a mixed catecholate-type siderophore) and pyochelin (a phenolate-type siderophore with lower iron affinity than pyoverdine), and these different siderophores might be differentially expressed depending on the environmental conditions because of non-redundant function in iron uptake (18, 64, 65). This was also observed for *Vibrio vulnificus* for which the catecholate siderophore was suggested to be important during human infections, whereas the hydroxamate siderophore may be more important in seawater (66), as well as for *Burkholderia cepacia* with strains isolated from the rhizosphere only producing a hydroxamate-like siderophore, while clinical isolates produced pyochelin and salicylate siderophores in addition to the hydroxamate-like siderophore (67). Differences in siderophore production were also previously reported among clinical strains as observed in the present study. Variable abilities in siderophore production were observed among *P. aeruginosa* clinical strains, with strains from patients with urinary tract and respiratory tract infections producing the highest levels of siderophores compared to those from patients with wound or burn infections (62, 68). In patients with CF, airway concentrations of total iron and ferritin-bound iron were shown to be higher, and these may enhance the growth of bacteria displaying a siderophore pathway leading to iron chelation, uptake, and utilization (69). Increased iron in the airways of these patients has also been considered as a factor favoring the persistence of bacterial infection (70).

In total, the results of this study support the fact that siderophores are important growth factors for *Achromobacter* spp., which may be essential to establishing infection in the host and surviving and persisting within the host. *A. xylosoxidans* showed distinct abilities in siderophore production, which warrant further investigations as they might well be related to the success of this species in human infections, particularly in patients with CF.

### Conclusion and outlook

This study represents the first ever evaluation of siderophore production by *Achromobacter* spp. on a large number of strains and accurately identified species. The capability of clinical and environmental strains to produce siderophores was demonstrated and their different capacities to produce siderophores, depending on the species under consideration and the origin of the strains, were elucidated. However, despite the substantial number of strains analyzed, it is still necessary to increase the number of isolates for certain rarely encountered species to more precisely evaluate their siderophore-production capacities. Several prospects have been opened up by the results of this study, first including a molecular analysis to identify both the type and potential diversity of siderophores produced by *Achromobacter* strains of different species and origins. Another question is whether *Achromobacter* spp. may capture siderophores produced by other bacteria (71), as well as the possible occurrence of modifications in siderophore production, which might be observed in CF patients throughout the length of airway colonization. Relating results with virulence in *in vitro* or *in vivo* models would also help to decipher the role of siderophore production in the host, as previously demonstrated for pyoverdine in *P. aeruginosa* (72). More broadly speaking, increasing the knowledge on the overall siderophore pathways in the *Achromobacter* genus, including biosynthesis, secretion, and uptake, as well as characterizing the factors favoring siderophore secretion (physicochemical and environmental factors, such as the effect of media composition, incubation time, etc.), is now required (25, 28). All this is particularly reinforced by the current development of research fields and therapeutic options aimed at limiting the iron available to the bacterial cell to reduce its multiplication and virulence. Indeed, over time, siderophores have not just been considered important virulence factors but are increasingly regarded as potential means of fighting against bacterial infections, by conjugating siderophores with antibiotics to generate “Trojan horse” antibiotics like cefiderocol or siderophore pathway inhibition (25, 26).

## MATERIALS AND METHODS

### *Achromobacter* spp. collection upstream of investigations

A total of 163 *Achromobacter* strains were included as follows: 67 CF strains from 67 CF patients, 61 NCF strains from 61 NCF patients, and 35 environmental strains (Table S3; Table 1). Clinical strains were collected during standard microbiological analyses performed as part of patients’ routine care. According to the type of sample analyzed, these analyses included a sample culture in blood culture vials or onto agar media including enriched media and/or selective medium for Gram-negative species like MacConkey agar. Strains isolated from lower respiratory tract samples (sputum, bronchoalveolar fluid lavage, distal airway secretions, and endobronchial aspirate) formed the majority and included all the 67 strains isolated from CF patients and 27 strains from NCF patients (44.3%). Other strains from NCF patients were from blood cultures and catheter (*n* = 14), eye and ear-nose-throat samples (*n* = 8), skin and soft tissue samples (*n* = 8), bone biopsy (*n* = 3), and rectal carriage (*n* = 1) (Table S3). Finally, 35 environmental strains were isolated either during bacteriological investigations in the domestic environment of CF patients (water, shower trap, and sink siphon) (*n* = 19) (3) or healthy individual (*n* = 1), from the hospital environment (water and surfaces) (*n* = 6), exterior locations (soil, rhizosphere, and water) in various countries (*n* = 8), or a nematode of the genus *Heterorhabditis* (*n* = 1) (Table S3). Most of them were recovered according to the procedure previously described by Dupont et al. (3) including a first *Achromobacter*-specific PCR-based screening step followed by cultivation onto an *Achromobacter* selective agar medium. All strains were stored frozen at −80°C.

### *nrdA* gene sequence determination, analysis, and phylogeny

*nrd*A genes were amplified as previously described (58) and sequenced on an ABI 3730xl automatic sequencer (Genewiz, France). *nrd*A sequences (765 bp) were compared to the PubMLST database (https:// pubmlst.org/organisms/achromobacter-spp) in order to determine the *nrdA* allele number. Maximum-likelihood (ML) analysis was performed using the NGPhylogeny website (https://ngphylogeny.fr/). The general time-reversible model was used as a substitution model. ML bootstrap support was computed after 100 reiterations. The data set for phylogeny analysis also included all the type strains of the *Achromobacter* species with validly published names and species with not validly published names, according to the List of Prokaryotic names with Standing in Nomenclature (https://lpsn.dsmz.de/genus/achromobacter), as well as representative strains of the genogroups available on the PubMLST database. The tree was formed by the online tool iTOL (https://itol.embl.de). All data generated during this study have been deposited in the PubMLST database.

### Siderophore quantification

Frozen strains were subcultured onto Trypticase-Soy (TS) agar and incubated at 37°C for 48 h. Pure culture of each strain was transferred from the agar medium to TS broth and allowed to multiply overnight at 37°C under aerobic conditions and shaken at 175 rpm. Strains of environmental origin were also tested after growth at 30°C for comparative analysis. The bacterial suspensions were adjusted to an optical density of 0.5 at 600 nm, corresponding to 2–6 × 10^8^ colony-forming units/mL. Global production of siderophores was quantified by the liquid chrome azurol-sulphonate (CAS) assay as described by Payne (73). Briefly, 20 µL of the calibrated bacterial suspension was cultured in 2 mL of iron-depleted minimal medium 9 for 48 h at 37°C. Culture supernatants were then added to a CAS-iron complex generated by adding a solution of iron hexahydrate (1 mM) to the CAS reagent (74). Based on the competition for Fe^3+^ between the CAS-iron complex and the bacterial siderophores having a higher affinity for Fe^3+^, the decomplexation of the CAS-iron complex is proportional to the amount of siderophores present in the culture supernatant tested and associated with a change in the color of the medium, from blue to orange, as measured at 630 nm. Inherent to its principle of siderophore detection and quantification, the CAS assay is a universal method that detects siderophores independent of their structure (75). Global siderophore production was quantified as percent siderophore units using the formula: [(negative control absorbance at 630 nm − sample absorbance at 630 nm)/(negative control absorbance)] × 100. According to Payne (73), a strain was classified as non-siderophore-producing if the psu value was below 10%. A clinical *P. aeruginosa* strain from a CF patient (*Pa* strain IV 33) was used as a positive control. For each strain, two technical replicates and two biological replicates were performed. Each value of siderophore dosage presented in this study is a mean value of four measures.

### Growth in iron-depleted minimal medium 9

Microtiter (96-well) plates (Nunc, Thermoscientific) containing 200 µL of MM9 were inoculated from overnight liquid cultures at an initial optical density at 600 nm (OD_600nm_) of 0.005. Plates were incubated at 37°C for 48 h without agitation in a microplate reader (Spark, Tecan). OD_600nm_ was measured every 5 minutes after a brief double orbital 2.5 amplitude agitation of 3 seconds for 48 h. Growth was expressed as the mean of five replicates.

### Statistical analysis

All the statistical tests were performed using GraphPad Prism (GraphPad Software, La Jolla, CA, USA). A two-tailed *P*-value < 0.05 was considered statistically significant (*P-value < 0.05, **P-value < 0.01, ***P-value < 0.001, and ****P-value < 0.0001).

A Shapiro-Wilk test was used to determine whether values followed a normal law. For variables not following a Gaussian distribution, nonparametric tests were carried out. To compare the ordinal qualitative and unpaired values (percentages of siderophore-producing strains in the overall population and depending on species and strain origins), a Chi-squared test was used. A Mann-Whitney *U*-test related to quantitative unpaired values was performed to compare the values for siderophore amounts in the overall population and depending on species and strain origins. Finally, the strength of the relationship between paired psu and OD_600nm_ values was statistically tested using the non-parametric Spearman’s correlation.

## SUPPLEMENTAL MATERIAL

Supplementary Figure S1 spectrum.02953-23-s0001.pdf Spearman's correlation between the amount of siderophores produced (psu) and growth in iron-depleted minimal medium 9 (OD_600nm_) for a selection of 33 *Achromobacter* spp. strains.

Supplementary Table S1 spectrum.02953-23-s0002.xlsx *nrdA* allele determined for the 163 strains of the study and species assignation according to strain origin.

Supplementary Table S2 spectrum.02953-23-s0003.xlsx Siderophore production in the genus *Achromobacter* according to species and strain origin.

Supplementary Table S3 spectrum.02953-23-s0004.xlsx Origin, identification and siderophore production for the 163 strains of the study, and complementary data (siderophore production at 30°C, growth in iron-depleted minimal medium 9) on subsets of strains.

ASM does not own the copyrights to Supplemental Material that may be linked to, or accessed through, an article. The authors have granted ASM a non-exclusive, world-wide license to publish the Supplemental Material files. Please contact the corresponding author directly for reuse.
