# Supplementary material for: Prevalence and variability of siderophore production in the Achromobacter genus
Source: Microbiol Spectr. 2024 Feb 5;12(3):e02953-23. doi: 10.1128/spectrum.02953-23 (PMC10913535; doi:10.1128/spectrum.02953-23)

Supplementary Figure 1. Spearman's correlation between percent of siderophore unit (psu) representing the amount of siderophores produced and optical dentity measured at 600 nm ($0D_{600nm}$) representing the growth in iron-depleted minimal medium 9 (MM9) for a selection of 33 *Achromobacter* spp. strains.

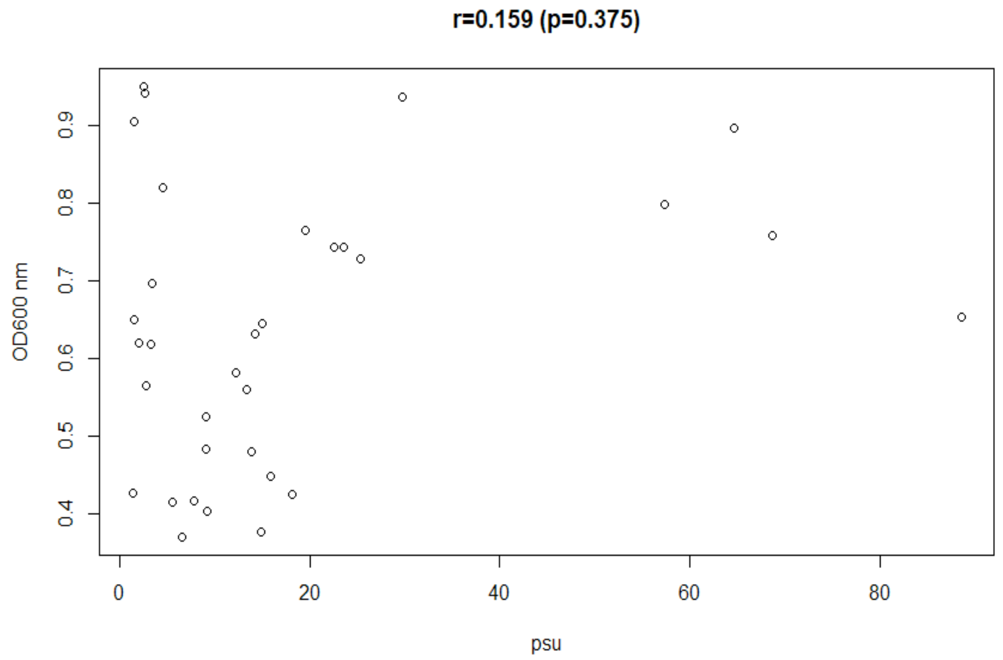

Supplement: Supplementary Figure S1 — Spearman's correlation between the amount of siderophores produced (psu) and growth in iron-depleted minimal medium 9 (OD600nm) for a selection of 33 Achromobacter spp. strains. [file spectrum.02953-23-s0001.pdf]
